# Severity and Mortality Predictors of COVID-19 Patients with Thrombotic Events-Evidence from the *“COVID-One”* Hospital in Albania

**DOI:** 10.3390/vaccines10111851

**Published:** 2022-10-31

**Authors:** Najada Como, Enkeleint A. Mechili, Migena Qato, Esmeralda Meta, Arjana Strakosha, Albana Fico, Albana Kenga, Athina E. Patelarou, Evridiki Patelarou

**Affiliations:** 1 “Mother Teresa” University Hospital, Dibra Street, 1000 Tirana, Albania; 2 Faculty of Medicine, Tirana University of Medicine, 1005 Tirana, Albania; 3 School of Medicine, University of Crete, 70013 Heraklion, Greece; 4 Department of Healthcare, Faculty of Health, University of Vlora, Kosova Street, 9401 Vlora, Albania; 5 Faculty of Nursing, Hellenic Mediterranean University, 71410 Heraklion, Greece

**Keywords:** COVID-19, D-dimer, CRP, coagulopathy, hospitalization, LDH, CK

## Abstract

COVID-19 vaccination leads to lower infection, morbidity, and mortality rates. However, COVID-19 infection leads to the development of coagulopathy-related manifestations in the form of both venous and arterial thromboembolism. This study aimed to assess the severity and mortality predictors of COVID-19 patients with thrombotic events in hospitalized patients in Albania. This is a retrospective study conducted in the “Mother Tereza” University Hospital of Tirana. Data were retrieved from the electronic databases of the hospital and only COVID-19 cases admitted to the infectious department during August–December 2020 were selected. Patients who, at admission, had a C-reactive protein (CRP) (mg/L) more than double and a D-dimer (ng/mL) more than triple according to international standards were included in the study. We performed univariate and multivariable logistic regression analysis, calculating unadjusted and adjusted odds ratios (ORs). A *p*-value < 0.05 was considered statistically significant. The study population included 60 hospitalized persons with a mean age of 64.4 years. Increased lactate dehydrogenase (LDH) (OR = 2.93; 95% CI = 0.82–10.42, *p*-value = 0.1) and increased creatine kinase (CK) (OR = 2.17; 95% CI = 0.63–7.46, *p*-value = 0.22) were related with increased probability of death. Moreover, a decreased number of lymphocytes was associated with increased mortality but with no statistical significance (OR = 0.40; 95% CI = 0.11–1.40, *p*-value = 0.15). The survival rate was higher for patients without comorbidities (*p* = 0.045). These results could serve as a baseline and as a reference for healthcare personnel who provides services to hospitalized patients with COVID-19. Further studies should take into consideration the vaccination of the population as well as including more hospitals and patients.

## 1. Introduction

Since December 2019, the globe has faced the COVID-19 pandemic, which started initially in China and since then quickly spread all over the world. As of 25 October 2022, more than 6.5 million deaths and more than 625,248,843 confirmed cases of COVID-19 have been reported globally [1]. A huge discussion has taken place about a possible vaccine for better management of the disease. After the onset of the pandemic, different companies started the development of various vaccines for COVID-19 and at present several vaccines are available for use [2]. These vaccines became a good and effective weapon in the management of the disease for decreasing infection as well as death rates.

European Medicines Agency (EMA) has authorized for use in Europe several vaccines, and their safety and efficacy have been proven [3,4,5]. All available vaccines up to now are reported to prevent severe disease, hospitalization, and death [6]. The efficacies of the BNT162b2 (third dose), NVX-CoV2373, and AZD1222 have been found to be 95.3%, 86.3%, and 64.3%, respectively [7,8,9]. In the unprecedented situation of the COVID-19 pandemic, governments applied mass-vaccination campaigns. At the moment vaccination is available with booster doses that have been mainly applied for the more vulnerable populations.

Despite the fact that new variants of the coronavirus are emerging, the use of authorized COVID-19 vaccines can decrease both morbidity and mortality rates [10,11]. According to a meta-analysis study, side effects after vaccination for cerebrovascular, cardiovascular, and allergic symptoms are low [12]. However, the authors report that cases of thrombosis are more likely in those vaccinated with viral vector vaccines [12]. The fear of thrombosis and other possible side effects were key reasons for not taking a COVID-19 shot. Additionally, other factors that contribute to vaccine hesitancy include demographic factors, beliefs about safety and effectiveness, level of education, beliefs in conspiracy theories, misinformation about the COVID-19 vaccine, etc. [13,14,15,16].

Daily clinical practice has already evidenced that the respiratory system is the most affected by the disease [17]. Acute respiratory distress syndrome is very common in patients with COVID-19 as well as being a major complication of the disease which leads to death [18]. Additionally, the most frequent late complications of COVID-19 disease are reported as lung injuries and venous/arterial thrombosis followed by other health problems [19]. One of the most common characteristics of patients with severe COVID-19 is coagulopathy. Development of coagulopathy-related manifestations in the form of both venous and arterial thromboembolism are also present after COVID-19 infection. Cytokine storm and cellular components contribute to thrombotic complications [20]. In the case of COVID-19, the cytokine storm seems more harmful and with a possible worse outcome, but the role of each cytokine is still not very clear [21].

The finding of biomarkers that can be used as key predictors of the disease has been one of the main tasks of health practitioners and researchers since the onset of the pandemic. High levels of D-dimer and C-reactive protein (CRP) have been reported as important indicators for coagulation dysfunction [22,23]. In general, CRP is considered a good predictor biomarker for the severity of the disease as well as its outcome [24]. A study concluded that both D-dimer and CRP are strongly correlated with higher levels of mortality and more cases of venous thromboembolism in COVID-19 patients [25].

As of 25 October 2022, 331,626 COVID-19 cases and 3592 deaths have been reported in Albania [26]. Recent data show that only 43.97% of Albanians are fully vaccinated [27]. Regardless of COVID-19, ischemic heart diseases and stroke are the two main causes of death in the country [28]. Health policymakers have made significant efforts to establish a preventive culture in Albania in the last few years [29]. However, to our best knowledge, there are no studies in the country that focused on thrombotic events during the COVID-19 pandemic in hospitalized patients. Additionally, a lack of studies in this field existed even before the pandemic situation. Due to the aforementioned, this study aimed to assess the severity and mortality predictors of COVID-19 patients with thrombotic events in hospitalized patients in Albania.

## 2. Materials and Methods

### 2.1. Setting

This is a retrospective study conducted in the University Hospital of Tirana “Mother Tereza” during the first phase of the COVID-19 pandemic (1 August–30 December 2020). The hospital is the biggest in Albania with a capacity of more than 1600 beds and around 2500 employees. Annually, it provides healthcare services to thousands of people not only from the capital Tirana but also from other parts of Albania. As the hospital is general, it has other wards including pediatric, emergency, surgery, intensive care unit (ICU), cardiology, anatomopathological laboratory, pathology, radiology wards, etc. This hospital also serves as a setting for medical students to conduct their practice as well as the only one that can conduct their residency after graduation. According to the Albanian Institute of Statistics, in 2020 in Albania, 201,449 were hospitalized while in the two previous years it was more than 275,000 people [30]. The “Mother Tereza” University Hospital of Tirana provides ambulatory services to around 300,000 people, hospitalization services to more than 80,000, and emergency services to more than 260,000 people annually [31].

During the COVID-19 pandemic, the hospital was the epicenter and provided healthcare services to those needed. It was named the COVID-one hospital, and the infectious disease department was reorganized totally in order to provide healthcare services to those diagnosed positive. People with other infectious diseases were transferred to alternative wards or facilities. The hospital hosted not just COVID-19 cases from Tirana City but also from all over the country patients received healthcare services from the hospital. This was because, during the first period of the pandemic, hospitals in the other cities were lacking the facilities and infrastructure. Moreover, the staff was unprepared for this unprecedented situation. The cases transferred to the COVID-one hospital were those that were moderate or serious and for whom receiving healthcare services at home was not effective. Patients were transferred to the COVID-one hospital from other regional hospitals or after communication with the National Emergency call center. After reorganization, the infectious diseases department had a capacity of 120 beds available for COVID-19 cases. On 2 January 2021 (the end date of the study), there were 58,991 total cases, 23,448 active cases, and 1190 deaths in Albania [32].

### 2.2. Data Extraction

Data extraction was conducted during the period of September–December 2021. Data were retrieved from the electronic databases of the hospital and only COVID-19 cases admitted to the infectious department during the period mentioned above were selected. During that period, 938 patients were admitted to the hospital. All patients admitted with SARS-CoV-2 infection were confirmed by using the standard method of real-time polymerase chain reaction (RT-PCR). In specific cases, additional examinations for confirmation of positive cases were conducted such as chest X-ray and/or computer tomography scan (CT). Data were extracted by two different researchers. They worked together for data extraction based on the criteria set initially. After admission at the hospital, patients undergo a series of health examinations based on their health status and the decision of their doctors or the doctor in charge at the moment of admission. Demographic characteristics (such as gender, age, and place of residence) and health history (such as the existence of chronic conditions, i.e., cardiovascular, diabetes, pulmonary, mental diseases, and malignancies) are recorded in the electronic databases. Additionally, information about days of hospitalization, clinical symptoms (such as temperature, sore throat, weakness, anosmia, anorexia, vomiting, diarrhea, dyspnea, and chest pain), and vital signs (cardiac rate/minute, respiratory rate/minute, blood pressure, and oxygen saturation in arterial blood) are available. Moreover, other information included in the database is the therapy provided (i.e., antibiotics or other kinds of medications provided), imaging (ultrasound, chest X-ray, CT), and laboratory findings (C-reactive protein (CRP) (mg/L), D-dimer (ng/mL)).

### 2.3. Inclusion and Exclusion Criteria

Initially, all patients admitted (the period August–December 2020) to the infectious disease department were extracted. After patients that at admission had a C-Reactive Protein-CRP (mg/L), more than double, and a D-dimer (ng/mL) more than triple according to international standards were selected. Normal levels of CRP (mg/L) are considered those lower than 10 mg/L [33] while for D-dimer (ng/mL), they are considered those lower than 500 ng/mL [34]. However, normal levels may range between laboratories. We set these two as key criteria since D-dimer is a key element for the prediction of vein thrombosis and mortality in patients with COVID-19 while CRP levels are a good predictor at admission for COVID-19 severity [35,36,37]. Additional inclusion criteria were all patients that manifested in a thrombotic event. We determined the time of the thrombotic event by referring to the day of symptoms onset (according to the patient’s medical history extracted from anamneses taken from patients themselves or from their relatives). Additionally, we divided the vascular accidents into two major groups according to topography: (1: upper limb and 2: lower limb). Patients that did not fulfill all the above criteria (or some data were missing) were excluded from the study. In total, after applying all inclusion/exclusion criteria, 60 patients were included. Moreover, we used the COVID-19 imaging score to evaluate the lung involvement in these patients. We considered three stages of lung involvement. The first stage considered all patients having pulmonary involvement score of 20–40%, the second stage all those with a range of 40–65% of pulmonary tissue affected, and the third stage all patients with more than 65% pulmonary involvement. We used this severity score system due to the fact that was simple, fast, calculated in real time during the CT examination, and did not need additional personnel, but was calculated automatically. Additionally, the hospital had a lack of kits and personnel focusing on this issue. None of the participants were vaccinated as the study finished before the onset of the mass-vaccination program.

### 2.4. Statistical Analysis

We use absolute numbers and percentages to present categorical variables. Additionally, we used the mean, standard deviation, median, interquartile range, minimum value, and maximum value to present continuous variables. Length of hospitalization and mortality were the dependent variables. The length of hospitalization did not follow a normal distribution and we transformed it into a dichotomous variable using the median as a cut-off point. Since the outcome variables were dichotomous, we used logistic regression analysis to identify the predictors of hospitalization and mortality. According to the Kolmogorov–Smirnov test, continuous variables did not follow the normal distribution. Thus, we used the median value to transform continuous variables into dichotomous variables to achieve a better function of logistic regression models. We performed univariate and multivariable logistic regression analysis, calculating unadjusted and adjusted odds ratios (ORs). Additionally, we calculated 95% confidence intervals (CI) and two-sided *p*-values. Moreover, we performed a log-rank test to estimate differences in mortality rates according to groups. A *p*-value < 0.05 was considered statistically significant. Statistical analysis was performed with the Statistical Package for Social Sciences software (IBM Corp. Released 2012. IBM SPSS Statistics for Windows, Version 21.0. Armonk, NY, USA, IBM Corp.).

### 2.5. Ethical Issues

All ethical issues were strictly followed in the study. No personal data were registered or collected. The study was approved by the Committee of Project Evaluation of the “Mother Teresa” University Hospital with Protocol Number 2425/1 dated 22 September 2021.

## 3. Results

The study population included 60 hospitalized persons with a mean age of 64.4 years old (standard deviation = 11, minimum age = 29, maximum age = 87). A total of 93.3% (*n* = 56) of the patients included were of male gender and 6.7% (*n* = 4) were females (Table 1).

**Figure 1:**
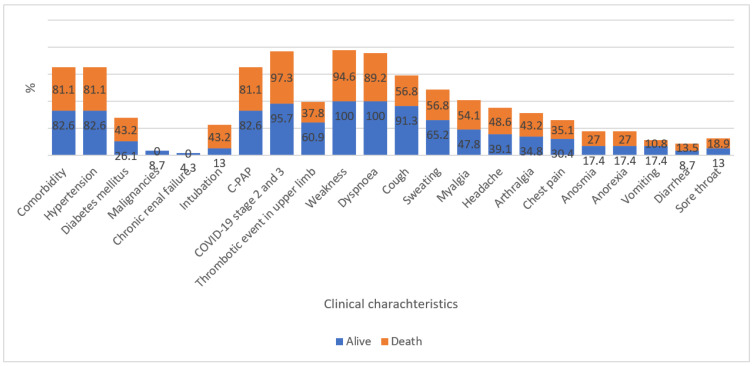
Comparison between survived and dead.

The clinical profile of patients is shown in Table 2. The mortality rate was 61.7% (37/60). The mean length of hospitalization was 9.3 days (standard deviation = 7.7, minimum = 1, maximum = 27), while median length was 8 days (interquartile range = 11). Comorbidity existed among 49 patients (81.7%). The most frequent diseases were hypertension and diabetes mellitus. Among patients, 26.7% were intubated and 26.7% were under C-PAP. As for the COVID-19 stages, 30.0% and 66.7% were at stages two and three, respectively. A total of 61.7% of thrombotic events happened in the lower limb. The most frequent symptoms and signs (Table 3) reported were weakness (96.7%), dyspnea (93.3%), cough (70%), sweating (60%), and myalgia (51.7%). Moreover, a graphic presentation of all indicators between survivors and deceased is presented in Figure 1.

Patients’ medication during their hospitalization is shown in Table 4. The most common pharmaceutical interventions in patients were levofloxacin (100%), heparin (100%), ceftriaxone (93.3%), lopinavir/ritonavir (63.3%), and methylprednisolone (50%). Descriptive statistics for the patients’ diagnostic tests are shown in Table 5. The mean value for ferritin was 2101 ng/mL; for lactate dehydrogenase (LDH), 738 U/L; for creatine kinase CK, 857 U/L; for fibrinogen, 578 mg/dL; for PCR-I, 169 mg/dL; and for D-dimer, 5748 mg/mL.

Univariate and multivariable logistic regression analyses with the length of hospitalization and death as the dependent variables are shown in Table 6. We did not find statistically significant relations in either case. However, we should notice that increased LDH (OR = 2.93; 95% CI = 0.82–10.42, *p*-value = 0.1) and increased CK (OR = 2.17; 95% CI = 0.63–7.46, *p*-value = 0.22) were related to an increased probability of death. On the other hand, a decreased number of lymphocytes was associated with increased mortality (OR = 0.40; 95% CI = 0.11–1.40, *p*-value = 0.15).

**Figure 2:**
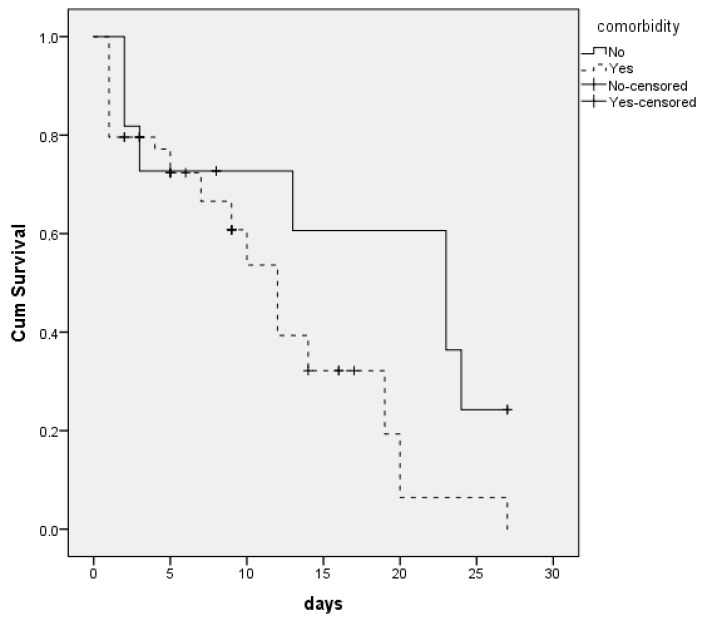
Survival curve for patients according to their comorbidities.

The survival rate was higher for patients without comorbidities (*p* = 0.045). In particular, the median survival rate for patients without comorbidities was 23 days (95% CI = 9.8–36.2), while for those with comorbidities was 12 days (95% CI = 9.6–14.4), (Figure 2).

## 4. Discussion

This study aimed to assess the severity and mortality predictors of COVID-19 patients with thrombotic events in hospitalized patients in Albania. Patients admitted to the biggest hospital in the country were selected for this study. From the multivariate analyses, we saw that high levels of LDH and CK were strongly associated with a higher possibility of death. Additionally, lymphopenia was statistically significantly correlated with high mortality rates. However, the presence of comorbidities was an indicator of a lower survival rate. The study results should be considered carefully as, since the first wave of COVID-19, different vaccines have been available which can decrease morbidity and mortality rates. The presence of these vaccines has improved the actual situation globally. In the current study, we did not include vaccinated people, and this should be studied in the future.

Thrombotic events are common in patients suffering from COVID-19 while few cases of thrombotic events have been reported after vaccination [38,39,40,41]. Different studies have focused on the possible association between vaccination for COVID-19 and thrombosis [39,40]. Even though there exists a slightly increased risk of a possible cerebral venous sinus thrombosis after vaccination with ChAdOx1, there is a significant benefit of vaccination for lower morbidity and mortality due to the disease [38]. Moreover, the benefit of vaccination is much higher than the possibility of a thrombotic event [42].

The results of this work are in line with previous studies that assessed COVID-19 severity and LDH levels. A study conducted by Henry et al. (2020), reached the conclusion that high levels of LDH are correlated with a six times higher probability of having a severe disease and 16 times higher mortality [43]. A systematic review and meta-analysis show that high LDH levels are correlated with a high probability of a negative outcome [44]. In a letter to the editor published in 2021, it was suggested that LDH can be used as a prognostic indicator for COVID-19 patients [45]. In the current study, patients with high levels of LDH are around three times more likely to die due to COVID-19 in comparison to those with lower levels. It is clear that healthcare practitioners should use LDH as a key biomarker during the provision of health services.

A study shows that increased levels of CK are strongly associated with more severity and higher mortality rates in COVID-19 patients [46]. A key result that emerged from the above study is that this indicator is independent and not affected by different sociodemographic factors or chronic health conditions. According to Kitbalian et al. (2021), CK levels are strongly correlated with fatal COVID-19 outcomes. Additionally, the authors report that CK should be used as an indicator for clinical outcomes, especially for men and those with diabetes [47]. Based on the current and previous studies we can say that the use of CK as a prognostic factor is important for patients with COVID-19.

Lymphopenia was another factor that was correlated with mortality rates in the current study. A cohort study in Korea reported that lymphopenia can serve as a predictive indicator not only for mortality but also for admission to the intensive care unit and the need for oxygen [48]. Patients with lymphopenia have high mortality rates according to a study conducted in Wuhan, while a review support that lymphopenia is associated with COVID-19 patients [49,50].

In contrast to other studies, in the current work, D-dimer did not show statistical significance with COVID-19 disease severity and/or negative outcomes. A study reports that D-dimer levels during hospital admission can serve as an indicator factor for mortality of the COVID-19 patients [51]. Another study shows that the use of D-dimer at a cut-off level of higher than 1.128 ng/mL is appropriate for predicting hospital mortality due to COVID-19 [52]. However, in the current work, we used different levels of D-dimer (>2425 vs. ≤2425). This, jointly with the low number of samples, most probably is the main factor for these differences. Further studies with a larger sample are strongly recommended.

The use of ferritin as a predictive indicator for patients with COVID-19 differs. According to a study published recently, levels of ferritin can be used as a layer for COVID-19 infection [53]. A study conducted in Israel reports that high levels of ferritin are correlated with COVID-19 severity [54]. The results of the current study did not show any statistically significant correlation between ferritin level and COVID-19 hospitalization and mortality. An Italian study concluded that ferritin is not correlated with disease outcomes in COVID-19 patients [55]. Despite the differences in the conducted studies, it is important for ferritin to be assessed jointly with the other laboratory indicators to guide patients’ treatment.

Different studies support that fibrinogen is a key predictor of COVID-19 severity [56,57]. The current study did not show such statistical significance. Similar to this, we did not find any correlation with gender. Most probably the low number of participants contributed to this result and further studies are needed to confirm or not this. Moreover, the presence of comorbidities was an indicator of a lower survival rate. A systematic review reached the conclusion that comorbidities are a good prognostic factor for patients with COVID-19 [58]. Another systematic review presents that people admitted to the hospital with comorbidities have an increased risk of mortality [59].

Vaccination of the population for COVID-19 should continue. Receiving booster doses is safe and effective for the population and can decrease severe disease [60]. Additionally, healthcare personnel can help significantly to raise awareness of the population for the benefits of vaccination. Working on misperceptions and fake news that circulate especially on social media is another critical issue in the continuous fight against COVID-19. Health policymakers should develop campaigns for the promotion of the safety and effectiveness of vaccines by focusing on different sociodemographic features of those who are skeptics.

### Strengths and Limitations

The current study suffers from some limitations. First of all, we used data from only one hospital and the sample size is small. Additionally, the lack of follow-up data is another weak point. Moreover, the retrospective nature of the study is also a limitation. Additionally, the sex ratio is unbalanced, and this is also a limitation of the current study.

However, despite the existence of some limitations, to our best knowledge, this is the first study in Albania that focused on assessing the severity and mortality predictors of COVID-19 patients with thrombotic events in hospitalized patients. Inclusion in the analysis of different factors such as laboratory examinations, health history, clinical symptoms, and imaging is a strong point of this work.

## 5. Conclusions

Finding key predictors for the severity and mortality of COVID-19 patients has been one of the most significant efforts done by practitioners and researchers in the last two years. Thrombotic events are common in COVID-19 patients and lead to longer hospitalization and mortality. According to the findings of the current study, high levels of LDH and CK are correlated with a higher possibility of mortality for hospitalized patients with COVID-19. The existence of lymphopenia could also be used as a key predictor. The current results could help healthcare personnel who offers services to hospitalized patients with COVID-19. This could help significantly in providing appropriate patient-centered services. Further studies should focus on including more hospitals and patients as well as a follow-up on data collection is strongly recommended. Additionally, a comparison between those vaccinated and non-vaccinated is strongly recommended.

## Figures and Tables

**Table 1:** Demographic characteristics of patients.

	N	%
Gender		
Male	56	93.3
Female	4	6.7
Age	Mean 64.4 (SD = 11)	
Place of residence		
Tirana	26	43.3
Durres	8	13.3
Vlore	3	5.0
Kavaje	3	5.0
Fier	10	16.7
Other	10	16.7

**Table 2:** Clinical profile of patients.

	N	%
Outcome		
Alive	23	38.3
Death	37	61.7
Comorbidity	49	81.7
Hypertension	49	81.7
Diabetes mellitus	22	36.7
Malignancies	2	3.3
Chronic renal failure	1	1.7
Intubation	16	26.7
C-PAP	16	26.7
COVID-19 stage		
Stage 1	2	3.3
Stage 2	18	30.0
Stage 3	40	66.7
Thrombotic event		
Lower limb	37	61.7
Upper limb	23	38.3

**Table 3:** Symptoms and signs of patients.

Symptoms and Signs	N	%
Weakness	58	96.7
Dyspnea	56	93.3
Cough	42	70.0
Sweating	36	60.0
Myalgia	31	51.7
Headache	27	45.0
Arthralgia	24	40.0
Chest pain	20	33.3
Anosmia	14	23.3
Anorexia	14	23.3
Vomiting	8	13.3
Diarrhea	7	11.7
Sore throat	10	16.7

**Table 4:** Patients’ medication during their hospitalization.

	N	%
Levofloksacin	60	100
Heparin	60	100
Ceftriakson	56	93.3
Lopinavir/ritonavir	38	63.3
Methylprednisolone	33	55.0
Dexamethazone	27	45.0
Cefepime	12	20.0
Albumine humane	10	16.7
Kabiven	8	13.3
Fresh frozen plasma	8	13.3
Plaquenil	8	13.3
Azytromicin	7	11.7
Aminoven	5	8.3
Vancomycin	4	6.7
Imipenem	5	6.7
Tigacil	4	6.7
Avigan	4	6.7
Meropenem	3	5.0
Tocilizumab	3	5.0
Metrondiazol	2	3.3

**Table 5:** Descriptive statistics for the patients’ diagnostic tests.

	Mean	Standard Deviation	Median	Minimum Value	Maximum Value
Ferritin (ng/mL)	2101	1304	1649	757	8171
PCR-I (mg/dL)	169	130	132	17	491
LDH (U/L)	738	550	511	237	2540
CK (U/L)	857	2174	106	16	9450
Fibrinogen (mg/dL)	578	177	570	274	1156
D-dimer (mg/mL)	5748	7181	2425	1160	25,231
Lymphocyte (%)	8.8	5.7	8	2	21

**Table 6:** Univariate and multivariable logistic regression analysis with the length of hospitalization (reference: length of hospitalization ≤ 8 days) and death (reference: alive) as the dependent variables.

Variable	Unadjusted OR ^1^ (95% CI)	*p*-Value	Adjusted OR ^1^ (95% CI)	*p*-Value	Unadjusted OR ^1^ (95% CI)	*p*-Value	Adjusted OR ^1^ (95% CI)	*p*-Value
Gender (males vs. females)	0.93 (0.12–7.08)	0.95			1.67 (0.22–12.73)	0.62		
Age (>64 vs. ≤64)	0.57 (0.21–1.60)	0.29			1.47 (0.51 0 4.24)	0.47		
Comorbidity (yes vs. no)	0.74 (0.19–2.74)	0.65			0.90 (0.23–3.50)	0.88		
COVID-19 stage (3 vs. 1–2)	0.91 (0.31–2.65)	0.86			1.52 (0.51–4.54)	0.45		
C-PAP (yes vs. no)	3.18 (0.94–10.72)	0.06			3.61 (0.90–14.48)	3.61		
Intubation **^2^** (yes vs. no)	2.19 (0.68–7.10)	0.19	0.91 (0.24–3.43)	0.89	NC	ΝC		
Thrombotic event (upper limb vs. lower limb)	1.28 (0.45–3.64)	0.64			0.95 (0.33–2.76)	0.92		
Ferritin (>1649 vs. ≤1649)	0.87 (0.31–2.39)	0.78			0.93 (0.33–2.63)	0.89		
PCR-I **^2^** (>132 vs. ≤132)	0.33 (0.10–1.08)	0.07	0.33 (0.10–1.08)	**0.07**	1.14 (0.36–3.62)	0.83		
LDH **^2^** (>511 vs. ≤511)	0.75 (0.27–2.11)	0.59			3.43 (1.12–10.47)	**0.03**	2.93 (0.82–10.42)	**0.10**
CK **^2^** (>106 vs. ≤106)	0.59 (0.20–1.74)	0.19			2.81 (0.91–8.67)	**0.07**	2.17 (0.63–7.46)	**0.22**
Fibrinogen (>570 vs. ≤570)	1.20 (0.30–4.82)	0.80			0.42 (0.10–1.72)	0.23		
D-dimer (>2425 vs. ≤2425)	1.14 (0.42–3.15)	0.80			0.65 (0.23–1.86)	0.43		
Lymphocyte **^2^** (>8 vs. ≤8)	0.97 (0.34–2.74)	0.95			0.29 (0.09–0.85)	**0.03**	0.40 (0.11–1.40)	0.15

^1^ An odds ratio < 1 indicates a negative association, while an odds ratio > 1 indicates a positive association. ^2^ In multivariable models, we included independent variables with a *p*-value less than 0.20 in univariate analysis. CI: confidence interval; NC: non-computable; OR: odds ratio.

## Data Availability

The data presented in this study are available on request from the corresponding author.
